# A Practical Résumé of Modern Methods Employed in the Treatment of Chronic Articular Osteitis of the Hip

**Published:** 1892-05

**Authors:** 


A Practical Resume of Modern Methods Employed in the Treatment of Chronic Articular Osteitis of the Hip. By Charles F. Stillman, M. Sc., M. D., Chicago. The Physician's Leisure Library. Detroit: George S. Davis. 1891. Price, cloth, 50 cents; paper, 25 cents.

There is no more important office that a physician can fulfil than to relieve a little sufferer from hip disease, and put such a child on his feet cured and ready to deal with the realities of life. We always feel a tenderness for, and an appreciation of, everything that the orthopedic surgeon does ; hence, have nothing but praise for his work, which is so often one of charity, and generally without compensation commensurate to the service performed. The book before us is a careful *résumé* of the progress that has been made in this department of surgery, and we commend it to the favor of the general practitioner, who may from it get an inspiration that will lead him to send his cases promptly to a competent orthopedic surgeon, and not wait until the favorable time has passed to arrest the disease without deformity or with only slight lameness.

